# Levothyroxine combined with selenium nanoparticles in one liposomal delivery system for hypothyroidism

**DOI:** 10.55730/1300-0527.3756

**Published:** 2025-08-05

**Authors:** Eylül ATAN, Güliz AK

**Affiliations:** 1 Department of Biochemistry, Faculty of Science, Ege University, İzmir, Turkiye; 2 Department of Molecular Medicine, Institute of Health Sciences, Dokuz Eylül University, İzmir, Turkiye

**Keywords:** Levothyroxine, selenium nanoparticles, hypothyroidism, liposomes, drug delivery

## Abstract

Levothyroxine, as a thyroid-stimulating hormone, is the primary drug used for hypothyroidism treatment, but it has poor bioavailability. Selenium (Se) plays an important role in regulating thyroid function; moreover, selenium nanoparticles (SeNPs) offer advantages over other Se forms. Liposomal delivery systems, especially those coated with polyethylene glycol (PEG), can increase the oral bioavailability of drugs and provide controlled release. In our research, as described in this paper, we aimed to develop an orally applicable liposomal delivery system to achieve enhanced treatment of hypothyroidism with SeNP characteristics, controlled levothyroxine release, and improved bioavailability of both drug and SeNPs. The SeNPs have been prepared using ascorbic acid with a hydrothermal method and an ecofriendly approach. The SeNPs have been 75–100 nm in size and have shown a Fourier transform infrared spectroscopy (FTIR) peak at 479 cm^−1^, which corresponds to the stretching and bending vibrations of the Se-O. Liposomes have been easily synthesized using the thin film technique, and levothyroxine and SeNPs have both been loaded by encapsulation. The encapsulation yield of levothyroxine into liposomes has been found to be 91.4%, which has been calculated spectrophotometrically. The SeNP content in the liposomes has been determined using inductively coupled plasma mass spectrometry (ICP-MS). The SeNPs and drug-encapsulated liposomes have been coated with PEG for potential oral usage, and their structure has been verified with FTIR. The levothyroxine release from the liposomal form has been higher in a pH 6.8 buffer than that in a pH 7.4 buffer, and the release has been observed to be in a controlled and constant manner with diffusion characteristics. Thus, we suggest that a PEG-coated liposomal delivery system containing both levothyroxine and SeNPs has been developed successfully, and it could be a promising approach for enhanced oral treatment of hypothyroidism.

## 1. Introduction

As a result of the thyroid gland’s inability to synthesize sufficient thyroid hormone, the body’s metabolic pathways are disrupted, resulting in the body’s failure to meet its metabolic needs. This leads to hypothyroidism, one of the most common chronic diseases. Untreated hypothyroidism can lead to serious adverse effects on organ systems and can contribute to hypertension, dyslipidemia, infertility, cognitive impairment, and neuromuscular dysfunction. Levothyroxine is the most commonly used drug as replacement therapy in patients with hypothyroidism [1,2]. Levothyroxine is a synthetic tetraiodothyronine (T4) hormone that is administered when the body’s natural hormone is insufficient and is biochemically and physiologically indistinguishable from the natural hormone [3]. The oral bioavailability of the drug is about 65%, which is reached when consumed 30 min to 1 h before breakfast. Moreover, the drug should be taken daily, and unfortunately, this needs the patient’s full cooperation with physicians and pharmacists. Hence, a formulation that is capable of sustained and controlled levothyroxine release could be a proper alternative for hypothyroidism in patients [4].

Liposomes were the first nanoparticulate drug delivery system to be studied and approved by the Food and Drug Administration (FDA) [5]. Liposomes are biocompatible carriers employed to improve the oral bioavailability of drugs, and they offer benefits derived from their lipidic bilayer structure. They can adhere well to biomembranes and form mixed-micelle structures with bile salts to increase the solubility of poorly soluble drugs. Stability under gastrointestinal conditions is the main concern for oral liposomes due to the pH changes that occur when injected, due to the bile salts, pancreatic enzymes, etc., in the gastrointestinal tract. However, the stability of liposomes can be improved by using appropriate lipid compositions, a polymer coating, and, e.g., stabilizing lipids. One of the most commonly used methods for surface modification of liposomes is the addition of hydrophilic polymers to the liposome surface. This method is also known as surface hydration. Polyethylene glycol (PEG) is generally used for this purpose. PEG coating can increase the circulation time of liposomes in the bloodstream or slow down their recognition by the reticuloendothelial system (RES) [6–9].

Selenium (Se) is a nonmetallic element, which, as an essential mineral for humans, has an important antioxidant effect that, in particular, forms selenocysteine, part of the active center of the glutathione peroxidase enzyme [10]. The human thyroid gland has the highest tissue Se content per gram of any human organ. Se has a systemic antiinflammatory effect and its deficiency affects free radical production, T4 to triiodothyronine (T3) conversion, and autoimmune processes, accelerating the symptoms of autoimmune thyroid disease [11]. The decrease in serum Se levels in patients with hypothyroidism proves that Se plays an important role in regulating thyroid function [12,13]. There are numerous studies regarding Se supplementation for hypothyroidism patients [14–16]. However, in general, conventional Se dietary supplements exhibit a low degree of absorption; moreover, Se is viewed as a controversial nutrient since high doses are toxic. Nanoscale size Se particles (5–200 nm) have gained popularity worldwide in recent years owing to their high degree of absorption, improved bioavailability, strong biocompatibility, higher bioactivity, and high efficiency in antiinflammatory, antioxidant, and immune-modulating properties compared with their organic and inorganic counterparts, while also being less harmful. Se nanoparticles (SeNPs) (the red, zero valent selenium or nanoselenium) have been widely researched in many medicinal uses ranging from antioxidant to anticancer effects due to their superior characteristics compared with other Se forms [17,18].

To our knowledge, our research described here is the first study that involved a PEG-coated liposomal delivery system carrying SeNPs and levothyroxine together for controlled and sustained drug release, oral administration, enhanced bioavailability, and improved bioactivity of a drug and selenium for hypothyroidism treatment. Although limited, some studies have investigated levothyroxine delivery for oral, injectable, or implantable systems, e.g., solid lipid nanoparticles [19], chitosan nanoparticles [20], poly D,L-lactic-co-glycolic acid-PEG-poly D,L-lactic-co-glycolic acid triblock system [4], poly(caprolactone)-based system [21], and poly (hydroxyethylmethacrylate-co-methacryloyl glutamic acid) nanocarrier system [22]. In a study reported by Aleskndrany and Şahin [23], the interactions of levothyroxine with lipids (dipalmitoyl phosphatidyl choline) in liposomes were demonstrated. In addition, there are a restricted number of works reporting SeNP carrying delivery systems, e.g., liposomal systems for increased bioavailability [7] and for targeted cancer therapy [24], and a silk fibroin coating system for thyroid cancer [25]. However, we know of no report on the combination of levothyroxine with Se or SeNPs in one delivery system for multidirectional treatment. Here, we aimed to develop an orally applicable liposomal delivery system to provide enhanced treatment of hypothyroidism, so there would be improved patient compliance and reduced costs. First, SeNPs were prepared using a nontoxic chemical, and then the liposomes were synthesized easily; levothyroxine and SeNPs were encapsulated in the liposomes, which were then coated with PEG for oral use. Controlled and sustained levothyroxine release was achieved with this formulation.

## 2. Materials and methods

### 2.1. Materials

L-ascorbic acid and sodium selenite (Na_2_SeO_3_) were purchased from Aromel Kimya Co., Corp. Levothyroxine sodium was donated by Abdi İbrahim İlaç Co., Corp. for use in the study. LIPOID P 75-3, Emulmetic 930, and cholesterol were purchased from Lipoid Kosmetics AG, Lucas Meyer Cosmetics Inc., and Sigma Aldrich Co. LLC, respectively. The 1,2-distearoyl-sn-glycero-3-phosphoethanolamine-N-[methoxy (polyethylene glycol)-2000] (DSPE-MPEG_2000_) was purchased from Biopharma PEG Scientific Inc.

### 2.2. Preparation and characterization of selenium nanoparticles

SeNPs were prepared using a modification of the method described by Shar et al. [26]. SeNPs were obtained by using L-ascorbic acid as a reducing agent for Na_2_SeO_3_. A total of 86.5 mg of Na_2_SeO_3_ was dissolved in 100 mL of distilled water and stirred at 80 °C for 1 h. Two grams of L-ascorbic acid were dissolved in 10 mL of distilled water and added dropwise to the Na_2_SeO_3_ solution while stirring with a homogenizer at 20,000 rpm. The color of the solution changed from light orange to dark orange during nanoparticle formation. Then the mixture was centrifuged at 12,000 rpm for 30 min, and the centrifuged pellet was washed with distilled water (d-water) three times. The SeNPs were dried in an oven at 60 °C overnight for characterization. The chemical structures of the dried SeNPs were determined by ultraviolet/visiblespectrophotometry (UV-VIS) (Cary UV Vis 60 spectrophotometer, Agilent Technologies Inc, USA), Fourier transform infrared spectroscopy (FTIR) (IRTracer-100, Shimadzu Corp, Japan), and X-ray diffraction (XRD) (PANalytical Empyrean, Malvern Inc, UK), and the morphological structures were investigated using scanning electron microscopy (SEM) (Apreo S, Thermo Scientific Inc, USA). The aqueous solution of SeNPs was analyzed by UV-VIS scanning, and dried particles were used for FTIR and XRD analyses. For SEM examination, dried nanoparticles were first covered with Au/Pd in a vacuum, then analyzed under a microscope.

### 2.3. Determination of levothyroxine with UV-VIS spectrophotometry

The levothyroxine calibration curve was obtained utilizing the report written by Rostami [20]. A 1-mg/mL levothyroxine sodium stock solution was prepared in ethanol. The prepared solution was sonicated for 20 min and then diluted to 100 μg/mL with sonicated d-water. The intermediate stock solution of levothyroxine was diluted to 20, 15, 10, 7.5, and 5 μg/mL with sonicated d-water and vortexed. The UV-VIS spectrophotometer was set at 227 nm, and a standard curve for levothyroxine was plotted using the absorbance values.

### 2.4. Levothyroxine encapsulation into liposomes and its characterization

The liposome synthesis was performed using a thin lipid film technique by modifying the methods described by Ahmed et al. [27] and Huang et al. [28]. To prepare the lipid film, 6 mg of Emulmetic 930, 6 mg of LIPOID P 75-3, and 1 mg of cholesterol were weighed and dissolved together in 2 mL of ethanol. The solution was kept in a water bath at 70 °C for 10 min with stirring. Next, the solvent was removed under N_2_ gas at 70 °C for the formation of a thin lipid film adhering to the walls of the glass tube. Two milliliters of pH 7.4 phosphate-buffered saline (PBS) were then added into the tube with the lipid film, covering the film, and kept in a sonic bath for 20 min. After that, an ultrasonicator homogenizer probe (Sonoplus, Bandelin Electronic GmbH & Co., Germany) was applied to the tubes for 3 min (55 s on, 5 s off, repeated 3 times), and the tubes were kept in the sonic bath, again, for 20 min. In order to load the drug into the liposomes, levothyroxine was added to the liposomes before the lipid film formation due to its hydrophobic structure. A total of 2 mg/mL of drug solution was prepared in ethanol. The lipids were weighed, dissolved in varying ethanolic drug solutions, and the lipid–drug solution was completed with ethanol to adjust the final volume to 2 mL. The final added drug concentrations were obtained as 1, 0.75, 0.5, and 0.25 mg/mL. The liposome formation procedure was applied to the tubes under the same conditions. At the end of the last sonication, the liposomes were dialyzed using a dialysis membrane (12,000–14,000 Da, Sigma Aldrich Co., LLC, USA) against d-water to remove the nonencapsulated drug for 2 h. Levothyroxine concentrations in dialysates were measured spectrophotometrically, and the encapsulation yields of the drug were calculated using Eq. 1. The levothyroxine-loaded liposomes (L-lipos) and also the empty liposomes were dried as films for characterization with SEM (JSM-7100-F, JEOL, USA) and FTIR (Spectrum Two, PerkinElmer, Inc., USA). The samples were prepared as mentioned in Section 2.2.


(1) $$drug\;encapsulation\;yield\;(\%)=\frac{amount\;of\;encapsulated\;drug\;(mg)}{initial\;amount\;of\;drug\;(mg)}\times 100$$

### 2.5. Preparation of liposomes loaded with levothyroxine combined with selenium nanoparticles and characterization

One milligram of SeNPs (synthesized as described in Section 2.2) was homogeneously dispersed in 10 mL of PBS using the ultrasonic homogenizer. The resulting 100 μg/mL SeNP dispersion was diluted 10-fold to 10 μg/mL. The lipids defined in Section 2.4 were dissolved in the optimum volume of ethanolic drug solution (0.75 mL), completed to 2 mL with ethanol, and, after solvent removal, 2 mL of SeNP dispersion (in PBS) was added to the liposomes. The sonication steps and dialysis were performed so the levothyroxine and SeNP-loaded liposomes (LS-lipos) were obtained. Selenium analyses of the liposomes were carried out using inductively coupled plasma mass spectrometry (ICP-MS) (7900 ICP-MS SPS4 Autosampler, Agilent Technologies Inc, USA) with acid pretreatment. The LS-lipos were also examined with FTIR.

### 2.6. mPEG coating of liposomes loaded with levothyroxine combined with selenium nanoparticles

For PEGylation of the LS-lipos, the postinsertion technique was utilized. DSPE-mPEG_2000_ in PBS was added to the LS-lipos based on the 10% w/w of the lipid content in LS-lipos [29,30]. The liposomes and the DSPE-mPEG_2000_ solution were mixed in a water bath set at 60 °C for 1 h under constant and mild stirring. At the end, the liposomes were kept at room temperature, and unintegrated DSPE-mPEG2000 was removed by a dialysis method for 2 h. The dried films of the PEGylated LS-lipos (LSP-lipos) were characterized with FTIR and SEM.

### 2.7. Drug release studies

LSP-lipos were tested in two different pH environments: pH 7.4 phosphate buffer to simulate the physiological pH, and pH 6.8 phosphate buffer for the gastrointestinal environment (n = 3). For levothyroxine release, 2 mL samples of LSP-lipo (containing 1436 μg of levothyroxine) were transferred to the dialysis membranes, and the membranes were placed in 10 mL of the related buffer. The membranes were shaken in a 37 °C water bath for 48 h. The released media were collected at predetermined time intervals (30 min–48 h) and changed with the same buffers under sink conditions. The released drug amount was analyzed, and the percentage of release was calculated according to Eq. 2 [31]. To compare the released data, the free drug release tests were also performed under the same conditions. For kinetic modeling, the release test data were analyzed, fitted to several kinetic release models (zero-order release, first-order release, Higuchi’s model, Korsmeyer–Peppas model, and Hixon–Crowell model), and the best fit model was determined based on the highest correlation coefficient (R^2^) value.


(2) $$cumulative\;release\;(\%)=\frac{total\;amount\;of\;released\;levothyroxine\;(\mu g)}{initial\;amount\;oflevothyroxine\;(\mu g)}\times 100$$

## 3. Results and discussion

### 3.1. Preparation and characterization of SeNPs

**Figure 1:**
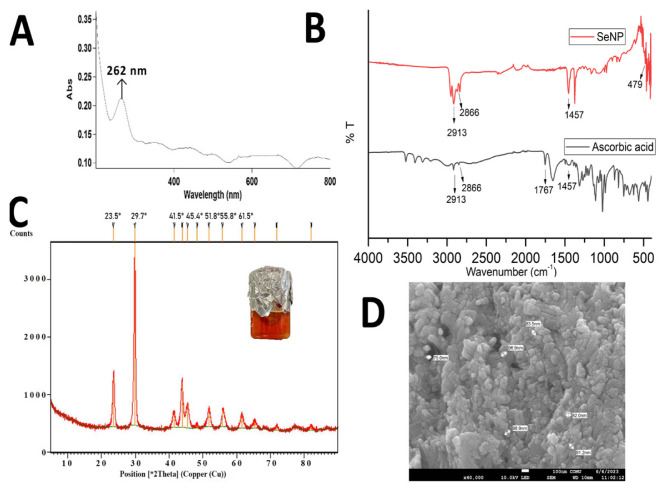
Characterization of SeNP: A) UV spectral scanning, B) FTIR spectrum (also of L-ascorbic acid), C) XRD analysis, D) SEM image.

One of the main methods for preparing SeNPs is the chemical reduction of Se salts with an ecofriendly approach. In this work, L-ascorbic acid was used as a nontoxic, biocompatible stabilizing agent. Selenite ions (4+) in the form of Na_2_SeO_3_ were easily reduced to zero valent Se by ascorbic acid using a hydrothermal method. By the end of the reaction, the SeNPs were formed and capped with dehydroascorbic acid. The reduction of Se ions to SeNPs by L-ascorbic acid was confirmed by the color change of the solution from light yellow to dark orange-red (brick red) [32]. The reason for the color change was the excitation of the surface plasmon vibrations of SeNPs. To determine and characterize the plasmon resonance of the synthesized SeNPs, UV spectral analysis was performed in the range of 200–800 nm (Figure 1A). The plasmon resonance peak associated with the crystallizability of the SeNPs is known to occur at a wavelength of 200–300 nm [26,33]. In our study, the peak at 262 nm corresponded to this characteristic peak of SeNPs.

The chemical structures of SeNPs and also free ascorbic acid were characterized by FTIR (Figure 1B). The peaks in the frequency range 3208–3524 cm^−1^, seen in the infrared (IR) spectrum of ascorbic acid, belong to different hydroxyl groups or the presence of moisture in the ascorbic acid sample used. In the IR spectrum of ascorbic acid, the broad peak at 1767 cm^−1^, which is due to the stretching vibrations in the lactone ring of ascorbic acid and is characteristic of ascorbic acid, disappeared in the IR of the SeNPs [34]. The sharp peak at 2913 cm^−1^ in the IR spectrum of the SeNPs is a peak belonging to the ether-methoxy-OCH_3_ groups. The aldehyde group at 2866 cm^−1^, the C=H vibration group at 1457 cm^−1^, and the secondary hydroxyl bond vibrations at 1257 cm^−1^ are seen in the IR diagram of ascorbic acid. The peak at 805 cm^−1^ represents C=O stretching vibrations and bending of the CH plane [26]. The IR spectrum of SeNPs shows that the peaks at 1637 cm^−1^ and 1618 cm^−1^ were weak and correspond to the asymmetric and symmetric vibrations of carboxylates, respectively, formed as a result of oxidation of the ascorbic acid adsorbed on selenium nanoparticles. It has been shown that the peak at 479 cm^−1^, which is specific to SeNPs and belongs to the stretching and bending vibrations of the Se-O bond, is related to the binding and interaction of SeNPs with carbonyl groups of ascorbic acid oxidation products [34]. Thus, the IR bands verified the SeNP structure, which was capped with ascorbic acid oxidation products. The phase diagram of the structure was obtained by XRD analysis, as shown in Figure 1C. The XRD phase diagram of SeNPs demonstrated sharp, fairly narrow peaks. The peaks at 23.5º, 29.7º, 41.5º, 45.4º, 51.8º, 55.8º, and 61.5º, which demonstrated the formation of a selenium crystalline phase, were considered as indicators of the successful formation of hexagonal SeNPs with a well-crystallized formation [35]. The morphology and dried particle size of SeNPs can be seen in Figure 1D. The shapes were nearly spherical, being between 75 nm and 100 nm, which is consistent with the literature [36].

### 3.2. Levothyroxine encapsulation into liposomes and its characterization

It is well known that liposomes have many advantages: liposomes provide long-term sustained release and produce less systemic toxicity. The barrier of the liposome structure has the ability to protect the drug or target from oxidation caused by gastric acid, alkaline solutions, or free radicals, compared with free drugs. All these reasons can be considered as indicators that liposomes are an ideal drug delivery system [37,38]. In this study, liposomes were chosen for oral levothyroxine delivery. Levothyroxine interacted with the lipids due to its hydrophobic feature, and then was encapsulated during the formation of the liposomes. The encapsulation efficiencies were calculated by measuring the nonencapsulated drug amounts spectrophotometrically (the obtained levothyroxine standard curve equation was y = 0.0212x with R^2^ = 0.9907). Table shows the initial drug concentrations and the encapsulation percentages obtained. The encapsulation value of the liposome initially loaded with 0.75 mg/mL of drug was above 90%. Our previous studies suggested that higher rates >90% are ideal for drug loading optimization [39,40]. A decrease in encapsulation yield was seen for the initial 1 mg/mL drug loading; thus, the optimal amount of drug for loading into liposomes was selected as 1436 μg of drug (corresponding to 0.75 mg/mL initially added drug concentration) with 91.4% yield.

**Figure 2:**
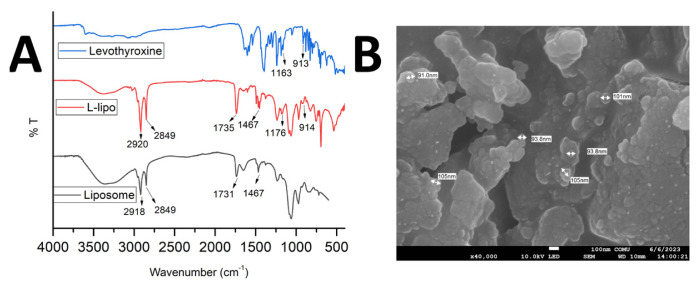
A) FTIR spectra of empty liposome, levothyroxine encapsulated liposome (L-lipo), and free levothyroxine sodium, B) SEM image of L-lipo.

Figure 2A shows the FTIR spectra of L-lipos (liposomes loaded with the optimized amount of levothyroxine), levothyroxine, and empty liposomes. The peaks at 1235 cm^−1^ (C-O stretching), 1467 cm^−1^ (C-H stretching), and 1645 cm^−1^ (N-H stretching) in the FTIR spectrum of the empty liposome (Figure 2A, black curve) structure are due to vibrations of the lipids. The peaks at 2918 cm^−1^ and 2849 cm^−1^ represent the symmetric and antisymmetric stretching vibrations of CH_2_, respectively. The peak at approximately 1731 cm^−1^ belongs to the stretching vibration of the C=O ester bond [41]. In the IR spectrum of levothyroxine (Figure 2A, blue curve), the stretching vibration peak of the phenolic O-H bonds is visible at 3595 cm^−1^. Similarly, vibrations from N-H bonds and O-H bands are indicated by the presence of multiple bands in the spectral range 3267–3595 cm^−1^. The peak at 1634 cm^−1^ is also associated with vibrations due to the N-H bonds. The stretching vibrations of the C-N bonds are related to the peak at 1054 cm^−1^, while the peak at 1534 cm^−1^ indicates the stretching vibrations of the C=C bonds arising from the aromatic components of the drug. The deformation peak of the bending vibrations of C-H bonds can be seen at 913 cm^−1^, while the in-plane deformation vibrations peak is visible at 1163 cm^−1^. Ledeti et al. [42] reported that the carboxylate anion exhibits an asymmetric stretching vibration of the C=O bond at 1579 cm^−1^ and a symmetric stretching vibration at 1394 cm^−1^. Additionally, a band at 1184 cm^−1^ indicates the vibrations of the C-O bonds, similar to those in our FTIR data, belonged to the drug. Upon comparison of the IR spectrum of L-lipos (Figure 2A, red curve) with the empty liposome (Figure 2A, black curve), certain different peaks were observed; specifically, the peaks at 1176 cm^−1^ and 914 cm^−1^ indicate the out-of-plane deformation vibrations of the C-H bonds, which come from the levothyroxine structure. In addition, the FTIR signals of L-lipo overlap with those of levothyroxine, which means successful encapsulation of levothyroxine into liposomes now. Figure 2B displays an SEM image of L-lipos. It can be concluded that the drug-loaded liposomes maintained their morphological similarity with the empty liposomes (data not shown), indicating that the addition of levothyroxine did not disrupt the liposome structure. It is worth noting that the particle sizes of our liposomes overlapped with those reported in a previous study [19].

### 3.3. Preparation of liposomes loaded with levothyroxine combined with selenium nanoparticles and their characterization

**Figure 3:**
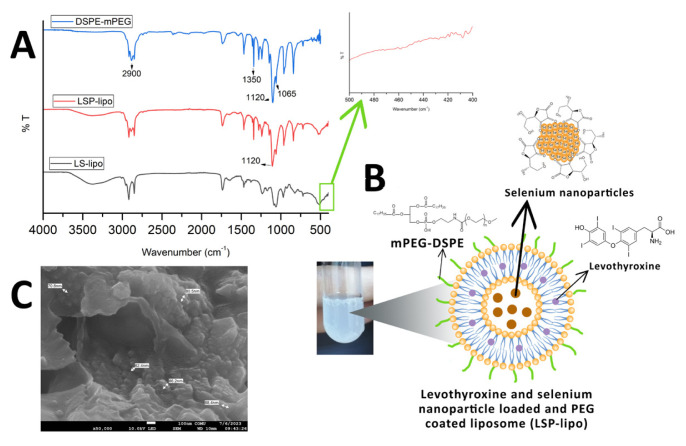
A) FTIR spectra of DSPE-mPEG, LS-lipo, and LSP-lipo; B) schematic display of levothyroxine and selenium nanoparticle-loaded and PEG-coated liposome (LSP-lipo); C) SEM image of LSP-lipo.

For enhanced hypothyroidism treatment, in our study, levothyroxine with SeNPs was aimed to be loaded in the liposomes. ICP-MS analysis was applied to determine the amount of selenium inside the liposomes. According to the obtained results, Se was present in the liposome at a concentration of 7.07 μg/mL. Moreover, in the FTIR spectrum of LS-lipos (Figure 3A), it can be determined, when zoomed in, that signals at 400–480 cm^−1^ correspond to Se-O bonds, indicating that SeNPs were incorporated into the liposome structure.

It can be inferred from the encapsulation data that 1436 μg of levothyroxine (described in Section 3.2) was loaded into liposomes together with approximately 14.14 μg of Se (in SeNP form), based on ICP-MS analysis. The obtained data values indicated that 130.55 μg levothyroxine and 1.29 μg Se (in SeNP form) were loaded per mg of liposomes. According to reported studies, the average dose of levothyroxine medication that a hypothyroidism patient should take is estimated to be approximately 25 μg per day [1,43]. In addition, the Se amount considered appropriate for supplementation in hypothyroidism patients should not exceed 400 μg/day [14], and it is also known that the bioavailability of Se from SeNPs is higher than from other Se forms. When the ratio of drug and selenium amount (in SeNP form) in one delivery system in our work was examined, it was possible to say that it is comparable with the general daily recommended drug and selenium intake range (also based on the release test data given in Section 3.5).

### 3.4. mPEG coating of liposomes loaded with levothyroxine combined with selenium nanoparticles

Liposomes prepared with phosphatidylcholine and cholesterol are referred to as traditional liposomes in the literature and are considered highly unstable in the bloodstream owing to opsonization by plasma proteins, leading to rapid clearance from circulation [44]. To minimize this issue, the PEGylation method has been utilized for over 30 years in clinical applications to optimize nanoparticulate drug delivery systems by enhancing their stability in the bloodstream, prolonging release kinetics, achieving high solubility, and reducing immunogenicity [45,46]. Furthermore, PEG coating minimizes the aggregation of orally administered nanoparticles in the gastrointestinal tract [47]. In this study, to develop a more stable liposomal drug delivery system, DSPE-mPEG_2000_ was incorporated into the structure through integration of the lipid chains of DSPE-mPEG via temperature change by movements (flip-flop and exchange movements) of phospholipids. The schematic illustration of LSP-lipos is shown in Figure 3B.

The PEG coating was confirmed by FTIR analyses. The FTIR spectra of DSPE-mPEG_2000_ and LSP-lipos are also displayed in Figure 3A. The peak at approximately 2900 cm^−1^ in the FTIR spectrum of DSPE-mPEG_2000_ represents CH_2_ stretching vibrations, while the peak at 1120 cm^−1^ indicates P-O stretching vibrations [28]. The signal at approximately 1350 cm^−1^ arises from C-N stretching vibrations, while the signal at approximately 1065 cm^−1^ corresponds to C-O stretching vibrations of ether and ester groups [48]. Particularly, the intensity of the peak at 1120 cm^−1^ in the IR spectrum of the PEG-coated liposomes was markedly increased compared to that in LS-lipos; we thus concluded the liposomes containing SeNPs and levothyroxine had been successfully coated with the DSPE-mPEG_2000_. Figure 3C shows an SEM image of the LSP-lipos. A slight decrease in size was seen in the morphology and size of LSP-lipos, as 62–88 nm, a similar decrease as compared to the size of L-lipos was also reported in a study describing size shrinkage of liposome caused by PEGylation by Wolfram et al. [49].

### 3.5. Drug release studies

**Figure 4:**
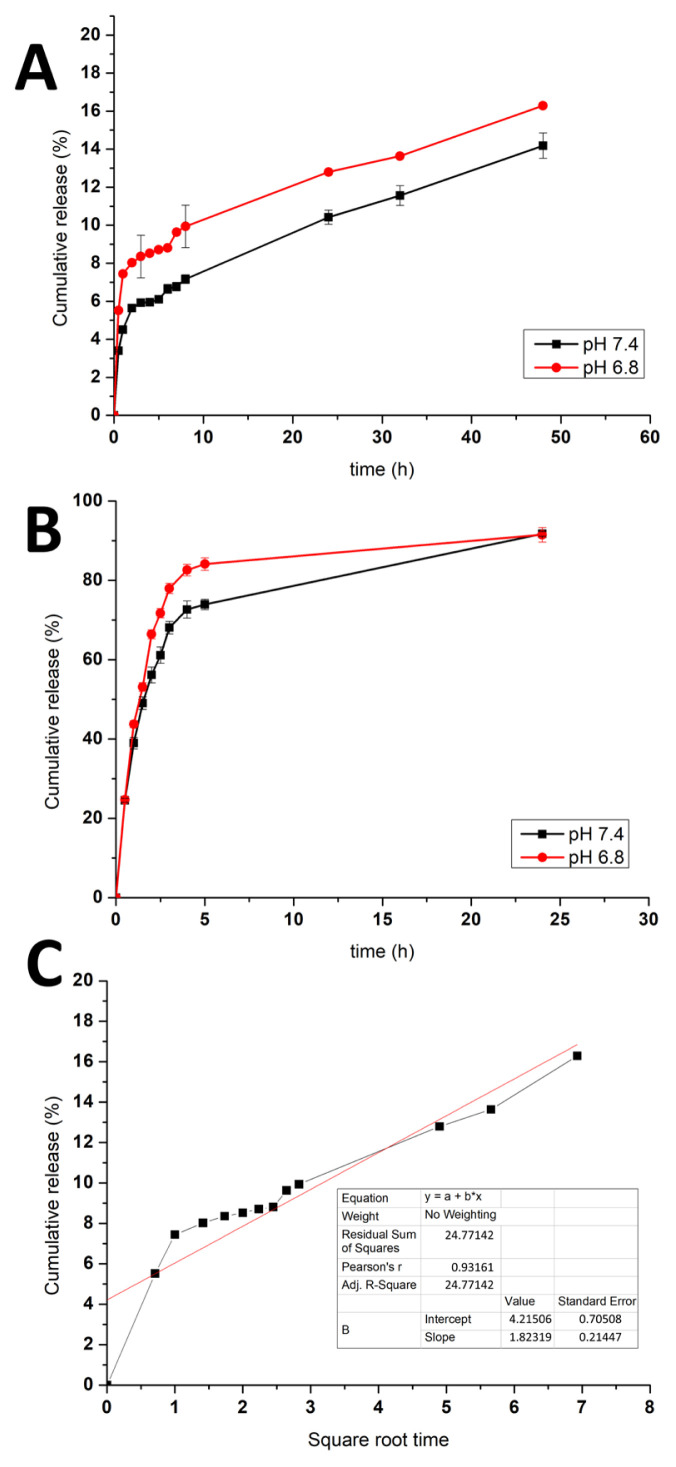
Release profile of levothyroxine from A) liposomal form, B) free form in both pH 7.4 and 6.8 buffers at 37 °C (n=3), C) Higuchi release kinetics profile of levothyroxine from the liposomal system.

The liposomes were subjected to release tests in a pH 7.4 physiological medium, and a pH 6.8 gastrointestinal medium, and all the data, maybe as cumulative release (%), are shown in Figure 4A. Furthermore, free levothyroxine release experiments were also conducted in pH 7.4 and pH 6.8 buffers to compare with the drug release between the PEGylated liposomes (shown in Figure 4B). The levothyroxine release degree from the liposomes in pH 7.4 was 5.64% while the degree was 8.3% in pH 6.8 at 2 h. At 48 h, the release percentage in pH 7.4 was still lower than that in the pH 6.8 buffer. The results implied that the drug release would be higher in gastrointestinal fluid than in physiological circulation, making more drug available for bioactivity in the colon in a controlled manner. In addition, by comparing the release kinetic modelling, the highest R^2^ value belonged to the Higuchi kinetic model (shown in Figure 4C), showing the drug release was diffusion-controlled and no burst release was obtained [50]. Nevertheless, the free drug release degree was higher than the release from the liposomal form, which was 56.15% in pH 7.4 and 66.42% in pH 6.8 buffer, and was almost completed at 24 h. It is suggested that liposomes provide controlled and sustained release of levothyroxine.

## 4. Conclusions

Hypothyroidism is a hormonal disorder that significantly affects human health. Nanomaterials are offering benefits for their treatment, e.g., eliminating the inconvenience of daily drug administration and improving patient compliance. In this study, levothyroxine and SeNPs were loaded together into liposomes, and the liposomes were PEGylated for enhanced stability. Characterization studies verified the assumed structure of the drug delivery system. In vitro drug delivery tests showed that a higher release degree was achieved in gastrointestinal fluid pH than physiological pH, with constant and controlled release throughout 48 h. The results demonstrated many advantages to conventional treatments, such as improving the hypothyroidism patients’ quality of life, ensuring their recovery without forgetting to take levothyroxine and selenium as a supplement daily, and also reducing the cost. Hence, our drug delivery system could be a promising approach for the alternative and innovative treatment of hypothyroidism.

## Figures and Tables

**Table:** Efficiencies of levothyroxine encapsulation into the liposomes. All values are the means ± SDs of measurements (n = 3).

Initial drug concentration (mg/mL)	Encapsulation efficiency (%)
0.25	76.7±1.5
0.5	86.5±1.2
0.75	91.4±0.9
1	86.0±0.8
